# Protective Effects of Withagenin A Diglucoside from Indian Ginseng (*Withania somnifera*) against Human Dermal Fibroblast Damaged by TNF-α Stimulation

**DOI:** 10.3390/antiox11112248

**Published:** 2022-11-15

**Authors:** Sullim Lee, Yea Jung Choi, Seulah Lee, Ki Sung Kang, Tae Su Jang, Ki Hyun Kim

**Affiliations:** 1 Department of Life Science, College of Bio-Nano Technology, Gachon University, Seongnam 13120, Republic of Korea; 2 College of Korean Medicine, Gachon University, Seongnam 13120, Republic of Korea; 3 Department of Oriental Medicine Biotechnology, College of Life Sciences, Graduate School of Biotechnology, Kyung Hee University, Yongin 17104, Republic of Korea; 4 School of Pharmacy, Sungkyunkwan University, Suwon 16419, Republic of Korea; 5 Department of Health Administration, Dankook University, Cheonan 31116, Republic of Korea

**Keywords:** *Withania somnifera*, withagenin A diglucoside, human dermal fibroblasts, TNF-α, skin aging

## Abstract

Human skin is constructed with many proteins such as collagen and elastin. Collagen and elastin play a key role in providing strength and elasticity to the human skin and body. However, damage to collagen causes various symptoms such as wrinkles and freckles, which suggests that they are important to maintain skin condition. Extrinsic or intrinsic skin aging produces an excess of skin destructive factors such as tumor necrosis factor (TNF)-α, which is a major mediator of the aging process. In aged skin, TNF-α provokes the generation of intracellular ROS (reactive oxygen species). It triggers the excessive secretion of MMP-1, which is a collagen-degrading enzyme that causes the collapse of skin collagen. Therefore, we aimed to search for a natural-product-derived candidate that inhibits the skin damage caused by TNF-α in human dermal fibroblasts. In this study, the protective effect of withagenin A diglucoside (WAD) identified from *Withania somnifera* against TNF-α-stimulated human dermal fibroblasts is investigated. *W. somnifera* (Solanaceae), well-known as ‘ashwagandha’, is an Ayurvedic medicinal plant useful for promoting health and longevity. Our experimental results reveal that WAD from *W. somnifera* suppresses the generation of intercellular ROS. Suppressing intracellular ROS generation inhibits MMP-1 secretion and the collapse of type 1 collagen. The effect of WAD is shown to depend on the inhibition of MAPK phosphorylation, Akt phosphorylation, c-Jun phosphorylation, COX-2 expression, and NF-κB phosphorylation. Further, WAD-depressed expression of the pro-inflammatory cytokines IL-6 and IL-8 triggers various inflammatory reactions in human skin. These findings suggest that WAD has protective effects against skin damage. Accordingly, our study provides experimental evidence that WAD can be a potential agent that can be applied in various industrial fields, such as cosmetics and pharmaceuticals related to skin aging.

## 1. Introduction

The skin is a large barrier of the human body and covers the human internal organs. The main components of the skin are the epidermis, the dermis, and subcutaneous tissues. The dermis is organized by an extracellular matrix (ECM) complex composed of collagen, elastin, and other protein components. Collagen is synthesized by fibroblasts in the dermis, and the ECM complex maintains flexibility, elasticity, and moisture in the skin [1]. Type 1 collagen is the major collagen protein produced in fibroblasts, accounting for over 90% of collagen [2]. The skin-aging process disrupts the collagen in the dermal ECM complex. Skin aging is largely divided into intrinsic aging and extrinsic aging. Intrinsic aging usually begins in the early 20s, but the visible symptoms do not appear on the skin immediately. On the other hand, extrinsic aging is caused by various external factors such as pollution and UV exposure, and symptoms are induced at an early stage. Particularly, UV radiation increases intracellular factors that accelerate skin aging such as reactive oxygen species (ROS) and the pro-inflammatory factor TNF-α [3,4,5]. TNF-α generates intracellular ROS and triggers a variety of inflammatory responses [6]. ROS upregulate the factors of skin aging such as matrix metalloproteinase-1 (MMP-1) and pro-inflammatory cytokines. MMP-1, which is known as collagenase, destroys collagen fibrils and damages the ECM [7]. Based on this background, we have been investigating natural products that can suppress TNF-α-induced human dermal fibroblast (HDF) damage [8,9].

*Withania somnifera* (L.) Dunal (Solanaceae), well-known as ‘ashwagandha’, is a medicinal plant; its leaves and roots have been used to promote longevity and optimal health in Ayurvedic medicine for a long time [10,11]. Due to the medicinal properties of *W. sominifera*, such as enhanced cognitive ability and stress release, the extracts of *W. sominifera* roots have been widely consumed as functional foods in the form of powder, tablets, and capsules. This famous medicinal plant has been intensively investigated in terms of the phytochemical constituents; withanolides were found to be the primary bioactive constituents of *W. sominifera* roots and exhibit diverse biological properties, such as neuroprotective, anti-inflammatory, immunomodulatory, anticancer, and antioxidant activities [12,13,14,15,16]. To date, more than 40 withanolides have been isolated from *W. somnifera*, including novel withanolides [17] as well as withanolide glycosides with β-D-glucopyranose linked at C-3 or C-27 [17]. According to our recent studies on *W. somnifera* roots, we reported bioactive new withanolides, namely withasilolides A–F [18] and withasomniferol D [19], which exhibit significant cytotoxicity against human cancer cells and anti-adipogenic activity. Specifically, a few withanolide glycosides have been reported in the group of withanolides, and a recent study reported that withanolide glycosides (withanosides I–XI) were found to show anti-Alzheimer, anti-stress, and neuroprotective activities [20]. Interestingly, some withanolide glycosides exhibited antiviral activity, which suggests that the active withanolide glycosides can be therapeutic agents against coronavirus disease (COVID-19) [20,21]. In our recent phytochemical study on *W. somnifera* roots, we found that withanolide glycosides from *W. somnifera* show anti-*Helicobacter pylori* and antioxidant effects [22]. Therefore, withanolide glycosides can be important candidates to exhibit various promising biological effects that contribute to health benefits.

As part of a continuing discovery of bioactive phytochemicals useful for health benefits [23,24,25,26,27], we examined a protective natural product from a methanolic (MeOH) extract of *W. somnifera* roots against human dermal fibroblast damage by TNF-α stimulation. To the best of our knowledge, no previous report has documented the ability of withanolide glycosides in *W. somnifera* to exhibit protective effects on TNF-α-exposed human dermal fibroblasts (HDFs). A phytochemical analysis of MeOH extracts of *W. somnifera* roots led to the isolation of one major withanolide glycoside, withagenin A diglucoside (WAD). We examined the inhibitory effects of WAD against TNF-α-mediated HDF damage and additional molecular signaling pathways. Herein, we describe the isolation and the structural elucidation of WAD and evaluate its protective effects against TNF-α-induced skin damage in HDFs and the underlying molecular mechanisms.

## 2. Materials and Methods

### 2.1. General Experimental Procedure and Plant Material

Detailed information regarding the general experimental procedure and the identification of the plant materials is provided in the Supplementary Material.

### 2.2. Extraction and Separation of WAD

Dried roots of *W. somnifera* (1.28 kg) were extracted with 80% MeOH for 3 days under reflux. The detained procedure for the isolation of WAD from the MeOH extract is provided in the Supplementary Material.

### 2.3. Cell Culture Condition

Human dermal fibroblasts (HDFs) were obtained from Promo cell GmbH (Heidelberg, Germany). HDFs were cultured with Dulbecco’s modified Eagle medium (Corning, Manassas, VA, USA). The media were supplemented with a 10% fetal bovine serum (Atlas, Fort Collins, CO, USA) and 100 U/mL penicillin-streptomycin solution (Gibco, Grand Island, NY, USA). The cell was incubated in a cell incubator with 5% CO_2_ humid in a 37 °C air condition.

### 2.4. Sample Preparation

WAD was dissolved in dimethyl sulfoxide (DMSO, Biosesang, Seongnam, Republic of Korea) to 10 mM for the cell treatment. TNF-α (PeproTech, Rocky Hill, NJ, USA) was dissolved in 1% BSA (Georgiachem, Norcross, GA, USA), and the final concentration of the cell treatment was 20 ng/mL.

### 2.5. Cell Viability Assay

HDF cells were seeded into 96-well plates, the density of cells was 0.5 × 10^4^ cells/100 μL in each well, and the seeded cells were incubated for 24 h. To arrest all cells in the same cycle, the medium was exchanged with a fresh medium without FBS (Fetal Bovine Serum) for 24 h. The cells were then treated with WAD at the indicated concentrations and incubated for 24 h. Then, 100 μL of a 10% EZ-cytox solution (DoGenBio, Seoul, Republic of Korea) was added per well and incubated in a cell incubator for 1 h. The cell viability of WAD on the HDFs was measured with a SPARK 10M device (Tecan Group Ltd., Männedorf, Switzerland) at a wavelength of 450 nm.

### 2.6. Intracellular ROS Generation Assay

HDF cells were seeded into 96-well black cell culture plates at 1 × 10^4^ cells/100 μL and were incubated in a cell culture incubator for 24 h. To evaluate the value at the same condition, the cells were starved in DMEM without FBS. Next, the HDF cells were exposed to 25, 50, and 100 µM WAD for 1 h and directly cotreated with a 20 ng/mL of TNF-α and 10 µM DCFDA (Sigma, St. Louis, MO, USA) mixture solution for 15 min. Then, the existing treatments were washed with phosphate-buffered saline using 100 µL per well (DPBS; Welgene, Gyeongsangbuk, Republic of Korea). The DCFDA-dyed HDF cells for intercellular ROS generation were evaluated using a microplate reader (SPARK 10M; Tecan) at the excitation and emission wavelengths of 485 nm and 535 nm, respectively. An IX51 fluorescence microscope (Olympus, Tokyo, Japan) equipped with a CCD camera was used to take photos of the DCFDA-dyed HDF cells.

### 2.7. ELISA Assay

HDFs were seeded into 48-well plates, and the density of the cells was 2 × 10^4^ cells/200 μL in each well. After this step, the cells were incubated in a cell incubator for 24 h prior to the ELISA assay. Then, cells were arranged in the same cycle by changing the old medium to a fresh medium without FBS. After 24 h, WAD was treated on the HDFs for 1 h and immediately exposed to TNF-α at 20 ng/mL. After 12 h, the supernatant was collected from the pretreated cells for the determination of the secretion of IL-6 and IL-8. Meanwhile, to measure the secretion of MMP-1 and procollagen I α1 (COLIA1), the supernatant was collected from the cells after 24 h. Secretion proteins were evaluated using a Human IL-6 Quantikine ELISA Kit (CAT No. D6050, R & D systems, Minneapolis, MN, USA), a Human IL-8/CXCL8 DuoSet ELISA (CAT No. DY208), a Human Total MMP-1 DuoSet ELISA kit (CAT No. DY901, R & D systems), and a Human Pro-Collagen I alpha 1 DuoSet ELISA (CAT No. DY6620-05, R & D systems). The optical density for protein secretion was measured using a SPARK 10M spectrophotometer (Tecan Group Ltd., Männedorf, Switzerland) with the wavelength set at 450 nm. The protein secretion results were calculated using a standard curve and are presented as fold-change values.

### 2.8. Immunoblotting Assay

HDFs were seeded into 6-well plates (Greiner, Thailand), and the number of cells was 3 × 10^5^ cells/2 mL in each well incubated progressively for 24 h. Then, the cells were arranged in the same cycle by changing the old medium to a fresh medium without FBS. After 24 h, WAD was added to the HDFs for 1 h, continuously exposed in the presence or absence of TNF-α (20 ng/mL). After 15 min, the cells were harvested to determine phospho-ERK (extracellular signal-regulated kinase), ERK, phospho-p38 (mitogen-activated protein kinases), p38, phospho-JNK (c-Jun N-terminal kinase), JNK, p-p65 (nuclear factor kappa B), p65, pc-Jun (Transcription factor Jun), c-Jun, and GAPDH. Meanwhile, the cells were harvested after 6 h to measure the expression of phospho-Akt (p-Protein kinase B), Akt (Protein kinase B), COX-2 (cyclooxygenase 2), HO-1 (heme oxygenase 1), and GAPDH. The cells were lysed with 120 μL of RIPA Buffer per well (Tech & Innovation, Gangwon, Republic of Korea). Afterward, to eliminate the cell pallets, the lysed cell was collected and spun down at 13,000 rpm at 4 °C for 30 min. After that, the supernatant was collected for protein samples. The supernatants containing the lysed protein were quantified using a BCA Protein Assay Kit (Thermo Fisher, Waltham, MA, USA). The primary antibodies phospho-ERK (CAT No. 9101S), ERK (CAT No. 9102S), phospho-p38 (CAT No. 4631S), p38 (CAT No. 9212S), phospho-JNK (CAT No. 9251S), JNK (CAT No. 9252S), phospho-Akt (CAT No. 4058L), Akt (CAT No. 9272S), p65 (CAT No. 4764S) and phospho-p65 (CAT No. 3033S), COX-2 (CAT No. 12282S), HO-1 (CAT No. 5061S), c-Jun (CAT No. 9165S), phospho-c-Jun (CAT No. 3270S), and GAPDH (CAT No. 2118S) were purchased (Cell Signaling Technology, Danvers, MA, USA). These antibodies were reacted with protein-sample-transferred PVDF membranes (MERCK, Darmstadt, Germany) for 12 h at 4 °C. The secondary antibodies, goat anti-Rabbit IgG-HRP (CAT No. 7074S, cell signaling), were reacted on the horizontal shaker for 2 h at room temperature. Protein bands were detected using a SuperSignal^®^ West Femto Substrate solution (Thermo Fisher Scientific, Waltham, MA, USA) and Fusion Solo Chemiluminescence imaging System 2 (PEQLAB Biotechnologie GmbH, Erlangen, Germany). The density of the detected bands was calculated based on the ratio to the GAPDH band. The analyzing results were represented using fold increase in comparison to the control group.

### 2.9. Statistical Analyses

The results of the experiment are estimated mean ± standard error of the mean (SEM) from duplicate and triplicate independent experiments. The differences in each experiment result were estimated using one-way analysis of variance (ANOVA), followed by Tukey’s honestly significant difference test (HSD). A *p*-value < 0.05 is assumed to be statistically significant.

## 3. Results

### 3.1. Isolation and Identification of Withagenin A Diglucoside (WAD)

**Figure 1:**
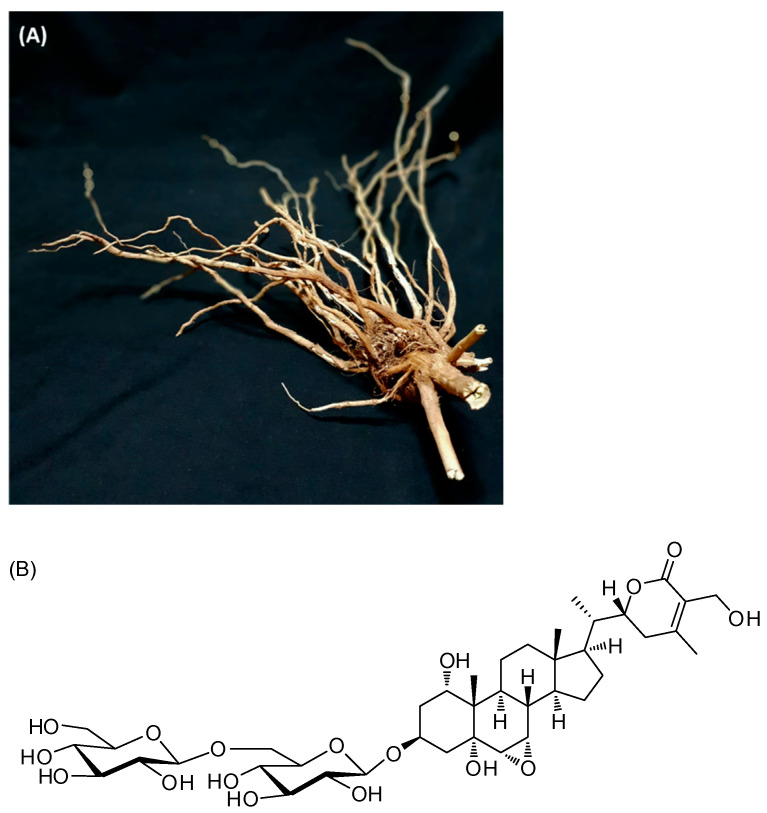
The photo of *W. somnifera* roots (**A**) and chemical structure of withagenin A diglucoside (WAD) (**B**).

The MeOH extract of the *W. somnifera* roots (Figure 1) was subjected to the solvent-partition process using four organic solvents—hexane, dichloromethane, ethyl acetate, and n-butanol—to afford four main fractions. An LC–MS analysis of the solvent-partitioned fractions reveals that the BuOH-soluble fraction has a major withanolide glycoside, which was isolated using repeated column chromatography and semi-preparative high-performance liquid chromatography (HPLC). The separation process resulted in the isolation of withagenin A diglucoside (WAD) (Figure 1), the structure of which was determined by conducting an analysis with nuclear magnetic resonance (NMR) spectroscopy and comparing its NMR spectroscopic data with those previously reported [28], along with MS data from the LC–MS analysis.

### 3.2. Effect of WAD on Viability of HDFs

**Figure 2:**
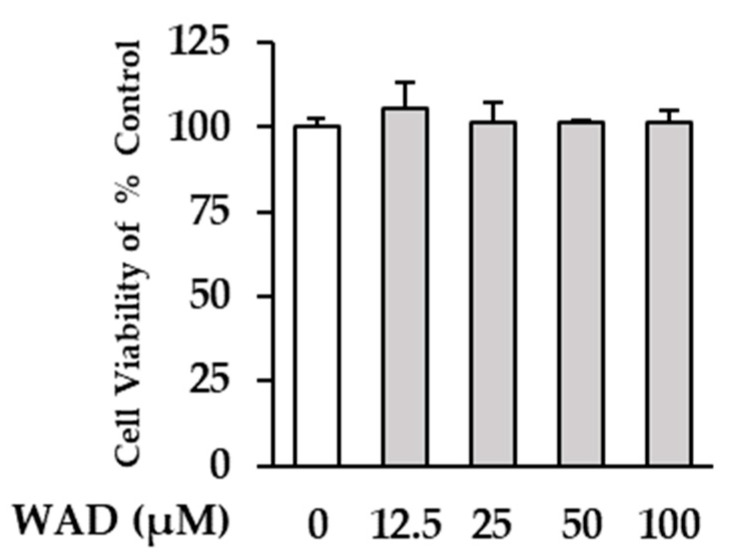
The effect of WAD on cell viability of HDFs. The cells were plated on 96-well cell culture plate with a density of 0.5 × 10^4^ cells/well and starved with fresh medium without FBS for 24 h. Next, WAD was treated on the seeded HDFs in the presented concentration for 24 h. The effect of WAD on HDF viability was evaluated using an Ez-Cytox kit. The results were displayed as mean ± SEM (*n* = 2).

In this study, we investigate the damage-inhibiting effect of WAD isolated from *W*. *somnifera* roots on human dermal fibroblasts (HDFs) exposed to TNF-α. Before assessing the effect of WAD, the cell viability of the compound itself against HDFs was measured. WAD was treated with the indicated concentrations of HDFs to determine cell viability. In Figure 2, the graph shows that WAD did not show significant cytotoxicity against HDFs up to 100 μM. Therefore, further experiments were performed with a concentration range up to 100 μM.

### 3.3. Effect of WAD on Intracellular ROS (Reactive Oxygen Species) Secretion in HDFs

**Figure 3:**
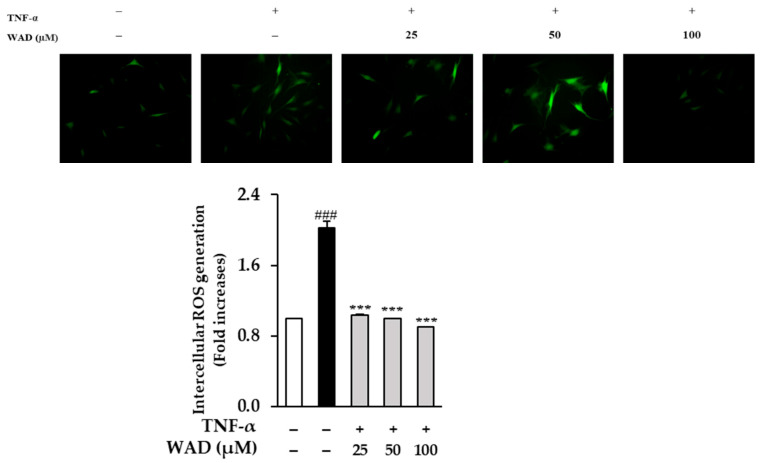
The effect of WAD on TNF-α-induced ROS generation in HDFs. The cells were seeded on 96-well black plates at concentration of 1 × 10^4^ cells/well and starved with fresh medium without FBS for 24 h. After that, the HDF cells were treated with the indicated concentrations of WAD for 1 h. Then, continuously, TNF-α (20 ng/mL) was added each well without control group. Next, the probe DCFDA (10 μM) was co-treated for 15 min. The fluorescence of DCFDA was determined using SPARK 10M plate reader at 485/535 nm. The fluorescence value was represented as mean ± SEM of duplicated experiments (*n* = 2). ^###^ *p* < 0.001 compared to non-treated group *** *p* < 0.001 compared to TNF-α-treated group.

The effect of WAD on the ROS secretion in HDFs exposed to TNF-α was investigated. As shown in Figure 3, the treatment of the HDFs with 20 ng/mL TNF-α resulted in a 2.02 ± 0.07-fold (*p* < 0.001) increase in intercellular ROS generation. In contrast, 25, 50, and 100 µM treatments of WAD reduced ROS generation 1.04 ± 0.01-fold (*p* < 0.001), 1.00 ± 0.01-fold (*p* < 0.001), and 0.9 ± 0.01-fold (*p* < 0.001), respectively.

### 3.4. Effect of WAD on MMP-1 and COLIA1 Protein Secretion in HDFs

**Figure 4:**
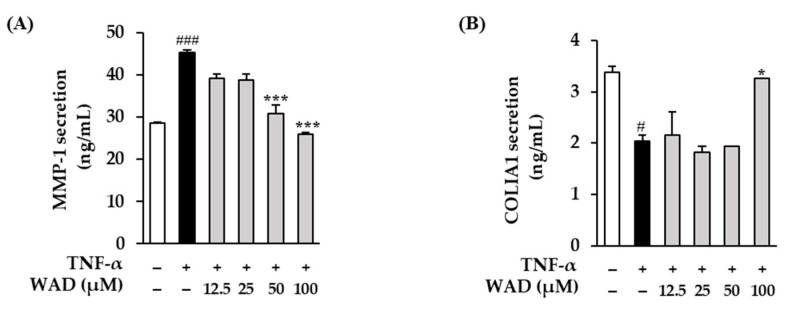
Effect of WAD on MMP-1 (**A**) and COLIA1 (**B**) protein secretion in TNF-α-stimulated HDFs). The HDFs were treated with 12.5, 25, 50, and 100 µM WAD for 1 h. After that, TNF-α (20 ng/mL) was added each well without control group for 24 h, continuously. The protein secretion levels (MMP-1 and COLIA1) were detected by using ELISA assay kits. The measuring results are represented as mean ± SEM of duplicated experiments (*n* = 2). ^#^ *p* < 0.05 and ^###^ *p* < 0.001 compared to non-treated group * *p* < 0.05 and *** *p* < 0.001 compared to TNF-α-treated group.

Next, we evaluated MMP-1 and COLIA1 secretion in the TNF-α-induced HDFs. TNF-α-induced MMP-1 secretion in the HDFs increases to 45.3 ± 0.60 ng/mL (*p* < 0.001) (Figure 4A). On the other hand, the 50 and 100 μM WAD treatments reduce it to 31 ± 2.05 (*p* < 0.001) and 26.0 ± 0.40 ng/mL (*p* < 0.001), respectively. COLIA1 secretion in the HDFs is reduced to 1.84 ± 0.10 ng/mL (*p* < 0.05) with the TNF-α treatment (Figure 4B). In contrast, it was significantly increased to 2.94 ± 0.00 ng/mL (*p* < 0.05) with the 100 μM WAD treatment.

### 3.5. Effect of WAD on Phosphorylation of MAPK Protein Expression in HDFs

**Figure 5:**
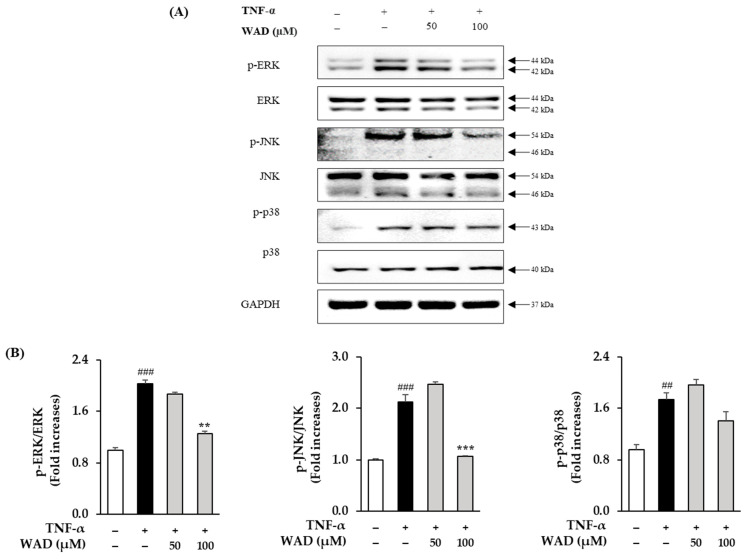
Effect of WAD on TNF-α-induced phosphorylation of MAPKs in HDFs. (**A**) WAD was treated on the HDFs for 1 h in the indicated concentrations. After that, TNF-α (20 ng/mL) was added each well without control group for 15 min. The MAPK expression bands were analyzed with Western blotting of p-c-Jun N-terminal kinase (p-JNK), JNK, p-ERK (p-extracellular signal-regulated kinase), ERK, p-p38, p38, and GAPDH. (**B**) The results of MAPK phosphorylation in comparison to control group were represented by the fold. The data are presented as mean ± SEM of triplicate experiments (*n* = 3). ^##^ *p* < 0.01 and ^###^ *p* < 0.001 compared to non-treated group and ** *p* < 0.01 and *** *p* < 0.001 compared to TNF-α-treated group.

We evaluated the effect of WAD on TNF-α-induced HDFs. As shown in Figure 5, an increase in ERK phosphorylation is observed in the HDFs exposed to TNF-α (2.0 ± 0.18-fold, *p* < 0.001). In contrast, the 50 and 100 μM WAD treatments decreased the phosphorylation of ERK 1.9 ± 0.02-fold and 1.3 ± 0.13-fold (*p* < 0.01), respectively. In the HDFs, the TNF-α treatment increased JNK phosphorylation 2.12 ± 0.15-fold (*p* < 0.001), and the 100 μM WAD treatment markedly decreased it 1.07 ± 0.01-fold (*p* < 0.001). The TNF-α treatment increased p38 phosphorylation in the HDFs 1.73 ± 0.11-fold (*p* < 0.01), and the 100 μM WAD treatment trended to decrease it 1.41 ± 0.14-fold.

### 3.6. Effect of WAD on Phosphorylation of c-Jun and NF-κB Protein Expression in HDFs

**Figure 6:**
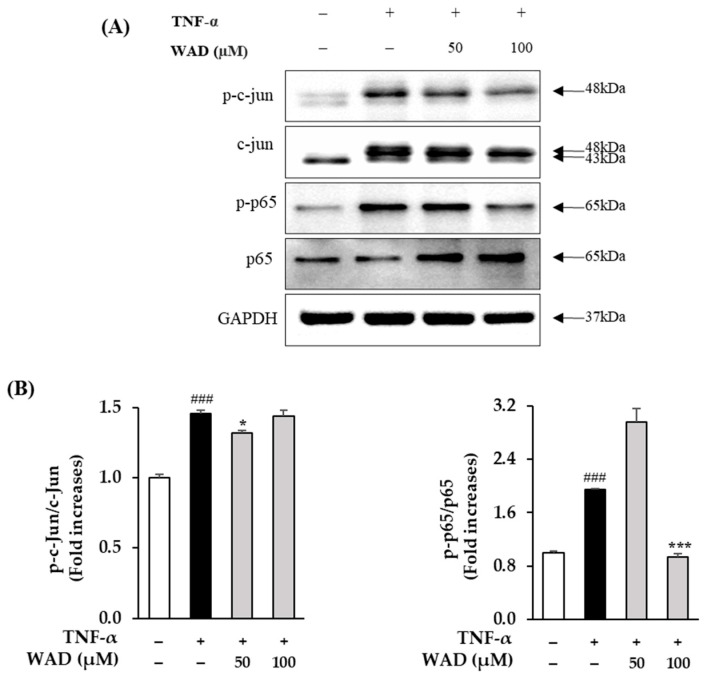
The inhibitory effects of WAD on NF-κB (p65) and c-Jun phosphorylation protein expression in TNF-α-induced HDFs. Specific concentration of WAD was treated on HDFs, and TNF-α (20 ng/mL) was added each well without control group for 15 min. (**A**) Protein expression bands of NF-κB phosphorylation, c-Jun, and GAPDH. (**B**) The expression of phosphorylation of NF-κB (p65) and c-Jun was measured using Western blot. The expression rates are described as mean ± SEM of triplicate experiments (*n* = 3). ^###^ *p* < 0.001 compared to non-treated group and * *p* < 0.05 and *** *p* < 0.001 compared to TNF-α-treated group.

Next, we investigated the inhibitory effect of WAD on the c-Jun and NF-κB expression in the HDFs exposed to TNF-α. In Figure 6, the TNF-α treatment increases the phosphorylation of c-Jun in the HDFs 1.46 ± 0.02-fold. On the other hand, 50 µM of WAD treatment slightly lessened it 1.32 ± 0.02-fold (*p* < 0.05). NF-κB phosphorylation in the HDFs increases with the TNF-α treatment 1.95 ± 0.01-fold (*p* < 0.001), whereas the phosphorylation of NF-κB significantly decreased it 0.94 ± 0.04-fold (*p* < 0.001).

### 3.7. Effect of WAD on Phosphorylation of Akt, COX-2, and HO-1 in HDFs

**Figure 7:**
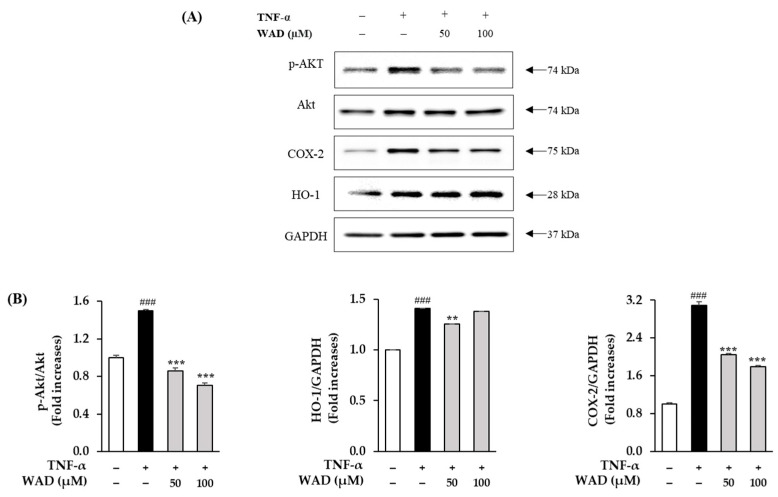
Effect of WAD on the phosphorylation of Akt and expression of COX-2 and HO-1 in TNF-α-induced HDFs. (**A**) WAD was treated with presented concentrations for 1 h. Next, TNF-α (20 ng/mL) was added each well without control group well for 6 h. The protein bands (phosphorylation of Akt [Protein kinase B], p-Akt, and expression of COX-2, HO-1, and GAPDH) were measured using Western blotting. (**B**) The protein expression and phosphorylation ratio are presented as fold-increase in comparison to non-treated group. The data are presented as mean ± SEM of triplicate experiments (*n* = 3). ^###^ *p* < 0.001 compared to non-treated group. ** *p* < 0.01 and *** *p* < 0.001 compared to TNF-α-treated group.

The experimental results show that the TNF-α treatment on the HDFs increased Akt phosphorylation 1.5 ± 0.01-fold (*p* < 0.001), whereas 50 and 100 μM WAD decreased it 0.86 ± 0.03-fold (*p* < 0.001) and 0.71 ± 0.03-fold (*p* < 0.001), respectively. The TNF-α-stimulated HDFs increased COX-2 expression 3.1 ± 0.07-fold (*p* < 0.001). On the other hand, the WAD treatment at 50 and 100 μM in the HDFs resulted in a 2.0 ± 0.02-fold (*p* < 0.001) and 1.8 ± 0.03-fold (*p* < 0.001) reduction, respectively (Figure 7). This result suggests that WAD can alleviate inflammatory activity in damaged skin. Heme oxygenase-1 (HO-1) has been reported to affect an antioxidant effect on HDFs [29]. In TNF-α-stimulated HDFs, HO-1 expression increases via the activation of the Nrf2 signaling pathway because of an increase in intracellular ROS. In our results, treatment with the indicated concentrations of WAD on TNF-α-stimulated HDFs did not significantly increase HO-1.

### 3.8. Effect of WAD on IL-6 and IL-8 Secretion in HDFs

**Figure 8:**
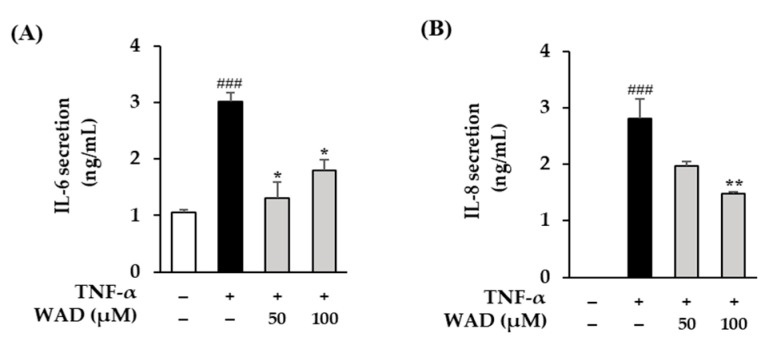
Effect of WAD on IL-6 and IL-8 protein secretion in TNF-α-induced HDFs. (**A**,**B**) WAD was treated on HDFs in specific concentrations for 12 h. Next, TNF-α (20 ng/mL) was added each well without control group well. The pro-inflammatory cytokine (IL-6 and IL-8) secretion was analyzed with ELISA assay using kits. The results are described as mean ± SEM of duplicate experiments (*n* = 2). ^###^ *p* < 0.001 compared to non-treated group and * *p* < 0.05 and ** *p* < 0.01 compared to TNF-α-treated group.

To evaluate whether WAD inhibits the cytokines secretion (IL-6, IL-8 and IL-1β) in TNF-α-induced HDFs, ELISA was conducted. In Figure 8A, the TNF-α treatment group on HDFs significantly increases IL-6 secretion to 3.02 ± 0.15 ng/mL compared to the control group (*p* < 0.001). Meanwhile, 50 and 100 µM of WAD treatment meaningfully decrease it to 1.30 ± 0.29 (*p* < 0.05) and 1.79 ± 0.20 ng/mL (*p* < 0.05), respectively. In Figure 8B, the TNF-α treatment group on HDFs significantly increases IL-8 secretion to 2.81 ± 0.34 ng/mL (*p* < 0.05) compared to the control group (*p* < 0.001). Meanwhile, 100 µM of WAD treatment decreases it to 1.48 ± 0.03 ng/mL (*p* < 0.01).

## 4. Discussion

The skin is the largest barrier in the human body and covers the human organs. The main components of the skin are the epidermis, the dermis, and subcutaneous tissue. The skin is made up of an ECM (extracellular matrix) complex including collagen, elastin, and other proteins. Collagen is the main component of the ECM, which accounts for 30% of the total protein in the human body [30]. Type 1 collagen is the main form of collagen in the skin [31]. Interstitial collagen is synthesized in fibroblasts and secreted as procollagen. Procollagen forms active collagen fibrils and maintains the tensile strength of the ECM [32]. Collagen degradation stimulates diverse aging diseases including skin aging, rheumatoid arthritis, and cardiac disease [33,34,35]. Thus, protecting against the degradation of collagen is crucial to maintaining a normal skin condition against skin-aging factors.

Skin aging is divided into two types: intrinsic and extrinsic aging. Intrinsic aging is dependent on time; however, extrinsic aging is developed by some external factors such as environmental pollution, severe physical stress, and exposure to UV radiation [36,37]. UV radiation plays a significant role in extrinsic aging, called photoaging. UV is harmful when continuously exposed to human skin [38]. UV-exposed skin accumulates ROS and damages DNA in the skin. The accumulation of ROS increases the secretion of collagenase, including MMP-1, and disrupts the collagen of the ECM [39,40]. Human skin exposed to UV expresses collagenases such as MMPs to destroy collagen [41]. About twenty types of MMPs have been identified, and these are secreted by skin cells such as keratinocytes and fibroblasts. MMPs hydrolyze the main components of the ECM and basement membranes such as collagen and elastin [42]. Among the MMPs, MMP-1 plays a vital role in destroying skin collagen [43]. This causes visual symptoms such as wrinkles and freckles on the skin [44]. We, therefore, evaluated the effect of WAD on MMP-1 and COLIA1 secretion in TNF-α-induced HDFs. The results in Figure 4 suggest that WAD can attenuate connective tissue damage via TNF-α-mediated MMP-1 inhibition and collagen reduction.

UV radiation is known to activate cell surface TNF-α receptors. This response triggers intracellular ROS and pro-inflammatory cytokines that are a major factor in both intrinsic and extrinsic aging [45]. ROS are produced by UVA and photosensitivity reactions and play a key role in skin aging. ^1^O_2_ (Single Molecular Oxygen) is a major type of ROS produced by the skin and causes various skin lesions such as acne and skin pigmentation [46]. Additionally, the overproduction of ROS is an essential mediator of photoaging and skin cancer [43]. ROS is mainly generated by mitochondrial respiration. The overgeneration of ROS degrades skin DNA molecules. DNA damage increases against reparability and initiates a tumor response in the skin [47]. We, therefore, investigated the inhibitory effect of WAD on TNF-α-induced ROS generation in HDFs. As shown in Figure 3, WAD inhibits intracellular ROS production in HDFs induced with TNF-α.

**Figure 9:**
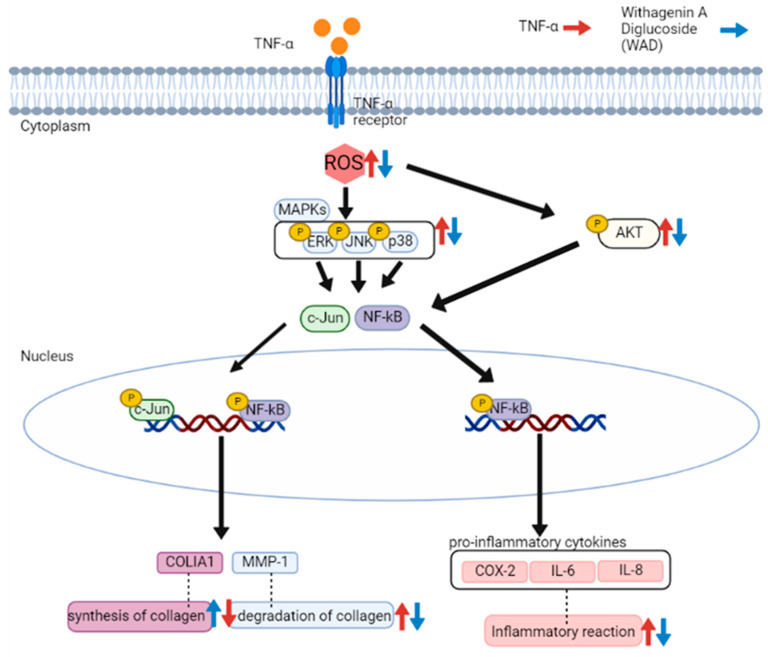
Schematic pathway illustrating the potential role of withagenin A diglucoside’s (WAD) protective effect in TNF-α-stimulated HDFs.

TNF-α-induced ROS generation regulates signaling pathways such as MAPKs and NF-κB. Specifically, ROS increase MAPK phosphorylation and activate the transcription factors AP-1 and NF-κB that regulate gene expression [48]. In detail, AP-1 is a complex of the transcription factors c-Fos and c-Jun. In particular, the JNK and p38 pathways are known to lead to AP-1 activation and c-Fos expression in UVA-induced HaCaT cells [49]. In Figure 5, WAD activation of AP-1 and NF-κB promotes the expression of MMP-1 and pro-inflammatory factors [50]. In addition, Akt (Protein kinase B)-associated ROS production controls diverse intercellular processes such as apoptosis and cell proliferation and differentiation. Akt stimulates NF-κB activation via IkB degradation [51]. In a previous study, the Akt-mediated phosphorylation of NF-κB was a critical upstream of inflammatory reaction in UVB-induced hairless mouse and HaCaT cells [52]. It has been reported that UV-activated Akt phosphorylation upregulates phosphorylation of NF-κB during skin aging [53]. NF-κB has been reported to regulate COX-2 and iNOS expression [54]. These inflammatory mediators have been studied that provoke the degradation of collagen, which increases the expression of MMPs such as MMP-1, MMP-3, and MMP-9 in dermal fibroblasts [55]. NF-κB, a heterodimeric complex of the redox-sensitive transcription factors p65 and p50, is a key modulator of COX-2 and iNOS. Thus, the inhibition of Akt phosphorylation and expression of COX-2 are important targets regarding TNF-α-mediated skin inflammation. In Figure 5, Figure 6 and Figure 7, WAD suppresses TNF-α-mediated HDF damage by inhibiting the phosphorylation of MAPKs, NF-κB, Akt, c-Jun, and the expression of COX-2. Furthermore, these transcription factors regulate the expression of various pro-inflammatory cytokines such as IL-6, IL-8, and IL-1β [56]. These cytokines induce a variety of skin lesions [57]. For example, both IL-6 and IL-8 may be involved in the formation of psoriatic lesions by promoting keratinocyte proliferation [58,59]. In Figure 8, WAD inhibits TNF-α-induced IL-6 and IL-8 hypersecretion in HDFs. These results suggest that WAD can protect TNF-α-induced damage, and we summarized the mechanism of the protective effect of WAD against TNF-α-induced HDF (Figure 9).

## 5. Conclusions

In conclusion, during the discovery study for a protective natural product against HDF damage by TNF-α stimulation, a phytochemical investigation of the MeOH extract of *W. somnifera* roots resulted in the isolation of a withanolide glycoside, withagenin A diglucoside (WAD). The structure of WAD was determined on the basis of NMR spectroscopic and ESIMS data. WAD is found to exhibit protective activity by inhibiting the intracellular ROS generation of HDFs stimulated with TNF-α. WAD protected the ECM by preventing an increase in collagenase and the destruction of collagen. Specifically, WAD suppressed the phosphorylation of MAPKs, Akt, NF-κB, and c-Jun, and the expression of COX-2. Furthermore, WAD inhibited the pro-inflammatory cytokines that provoke the inflammatory responses associated with skin aging. Although further studies need to be conducted, these results suggest that WAD can be considered a potential protective agent against the TNF-α-induced skin-aging process. In this study, we only evaluate the protective effect of WAD against skin aging in HDF cells. Further studies of in vivo models are needed to fully understand the skin anti-aging effects of WAD. Another study found that fermented blackberry inhibits aging through the modulation of MAPK/NF-κB signaling in UV-exposed hairless mouse and human foreskin fibroblasts (Hs68) [60]. In addition, human collagen alpha-2 type I induces collagen synthesis, wound healing, and elastin production in HDFs [61]. Therefore, the protective effect of WAD on the inhibition of UV-stimulated collagen synthesis will be demonstrated in future studies.

## Data Availability

The data presented in this study are available on request from the corresponding author.

## Supplementary Materials

The following are available online at https://www.mdpi.com/article/10.3390/antiox11112248/s1: General experimental procedure; Figure S1: ^1^H NMR (850 MHz) data for WAD in CD_3_OD.
