# Supplementary material for: Protective Effects of Withagenin A Diglucoside from Indian Ginseng (Withania somnifera) against Human Dermal Fibroblast Damaged by TNF-α Stimulation
Source: Antioxidants (Basel). 2022 Nov 15;11(11):2248. doi: 10.3390/antiox11112248 (PMC9686661; doi:10.3390/antiox11112248)
Supplement: Supplementary file 1 [file antioxidants-11-02248-s001.zip › antioxidants-1955670-supplementary.pdf]

Supplementary data

Protective Effects of Withagenin A Diglucoside from Indian Ginseng (*Withania somnifera*) against Damage by TNF-α stimulation in Human Dermal Fibroblasts

Sullim Lee [1,†], Yea Jung Choi [2,†], Seulah Lee [3,4,†], Ki Sung Kang [2], Tae Su Jang [5,*] and Ki Hyun Kim [4,*]

[1] Department of Life Science, College of Bio-Nano Technology, Gachon University, Seongnam 13120, Republic of Korea
[2] College of Korean Medicine, Gachon University, Seongnam 13120, Republic of Korea
[3] Department of Oriental Medicine Biotechnology, College of Life Sciences, Graduate School of Biotechnology, Kyung Hee University, Yongin 17104, Republic of Korea
[4] School of Pharmacy, Sungkyunkwan University, Suwon 16419, Republic of Korea
[5] Department of Health Administration, Dankook University, Cheonan 31116, Republic of Korea
* Correspondence: jangts@dankook.ac.kr (T.S.J.); khkim83@skku.edu (K.H.K.); Tel.: +82-41-550-1476 (T.S.J.); +82-31-290-7700 (K.H.K.)
[†] These authors contributed equally to this work.

Supporting Information Contents:.

General experimental procedure……………………………………………………………………………………………..…….S3
Figure S1. ^{1}H NMR (850 MHz) data for WAD in CD_3OD………….…………………………………...………………………………S5

General experimental procedure

Optical rotations were measured using a JASCO P-2000 polarimeter (JASCO, Easton, MD, USA). Ultraviolet (UV) spectra were acquired on an Agilent 8453 UV-visible spectrophotometer (Agilent Technologies, Santa Clara, CA, USA). Electronic circular dichroism (ECD) spectra were measured on a JASCO J-1500 spectropolarimeter (JASCO). Infrared (IR) spectra were recorded with a Bruker IFS-66/S FT-IR spectrometer (Bruker, Karlsruhe, Germany). Nuclear magnetic resonance (NMR) spectra were recorded with a Bruker AVANCE III HD 800 NMR spectrometer with a 5 mm TCI CryoProbe operating at 850 MHz (^{1}H) and 212.5 MHz (^{13}C), with chemical shifts given in ppm (δ) for ^{1}H and ^{13}C NMR analyses. Preparative high-performance liquid chromatography (HPLC) was performed using a Waters 1525 Binary HPLC pump with a Waters 996 Photodiode Array Detector (Waters Corporation, Milford, MA, USA) and an Agilent Eclipse C18 column (250 × 21.2 mm, 5 μm; flow rate: 5 mL/min; Agilent Technologies). Semi-preparative HPLC was performed using a Shimadzu Prominence HPLC System with SPD-20A/20AV Series Prominence HPLC UV-Vis detectors (Shimadzu, Tokyo, Japan) and a Phenomenex Luna C18 column (250 × 10 mm, 5 μm; flow rate: 2 mL/min; Phenomenex, Torrance, CA, USA). LC/MS analysis was performed on an Agilent 1200 Series HPLC system equipped with a diode array detector and 6130 Series ESI mass spectrometer using an analytical Kinetex C18 100 Å column (100 × 2.1 mm, 5 μm; flow rate: 0.3 mL/min; Phenomenex). Silica gel 60 (230-400 mesh; Merck, Darmstadt, Germany) and RP-C18 silica gel (Merck, 230-400 mesh) were used for column chromatography. The packing material for molecular sieve column chromatography was Sephadex LH-20 (Pharmacia, Uppsala, Sweden). Thin-layer chromatography (TLC) was performed with precoated silica gel F254 plates and RP-C18 F254s plates (Merck) and spots were detected under UV light or by heating after spraying with anisaldehyde-sulfuric acid.

Plant material

One-year old roots of *W. somnifera* were purchased from Seong-geo-san Farm, Cheonan, Korea in October 2016, and the plant was identified by one of the authors (K. H. Kim). A voucher specimen of the material (IDG-2016) was deposited in the herbarium of the School of Pharmacy, Sungkyunkwan University, Suwon, Korea.

Extraction and separation of the compounds

Dried roots of *W. somnifera* (1.28 kg) were extracted using 80% aqueous MeOH (3.0 L) for 3 days under reflux, performed three times, and filtered at room temperature. The filtrate was concentrated using a rotavapor to obtain the MeOH extract (189.6 g), suspended in water (700 mL) and partitioned with 700 mL of four solvents: Hx, MC, EA, and *n*-BuOH. The four fractions were obtained in the following order: Hx (3.4 g), MC (4.5 g), EtOAc (2.0 g), and *n*-BuOH-soluble (18.6 g) fractions. These four fractions, obtained from solvent partitioning, were

examined by LC/MS analysis. The LC/MS analysis revealed the presence of one major withanolide glycoside in the *n*-BuOH-soluble fraction. The *n*-BuOH-soluble fraction (18.6 g) was subjected to silica gel open column chromatography (150 g, eluted with CH_2Cl_2/MeOH [30:1 → 1:1]), to afford six fractions (B1–B6). Fraction B6 (340 mg) was subjected to reverse-phase (RP) silica gel open column chromatography with MeOH/H_2O (4:6 → 10:0), yielding four subfractions (B6a-B6d), and subfraction B6c (150 mg) was subjected to preparative HPLC (65% MeOH → 80% MeOH, gradient solvent system) to yield three subfractions (B6c1-B6c3). Subfraction B6c3 (60 mg) was separated by semi-preparative HPLC with 49% MeOH to obtain WAD (t_R 60.0, 10.2 mg).

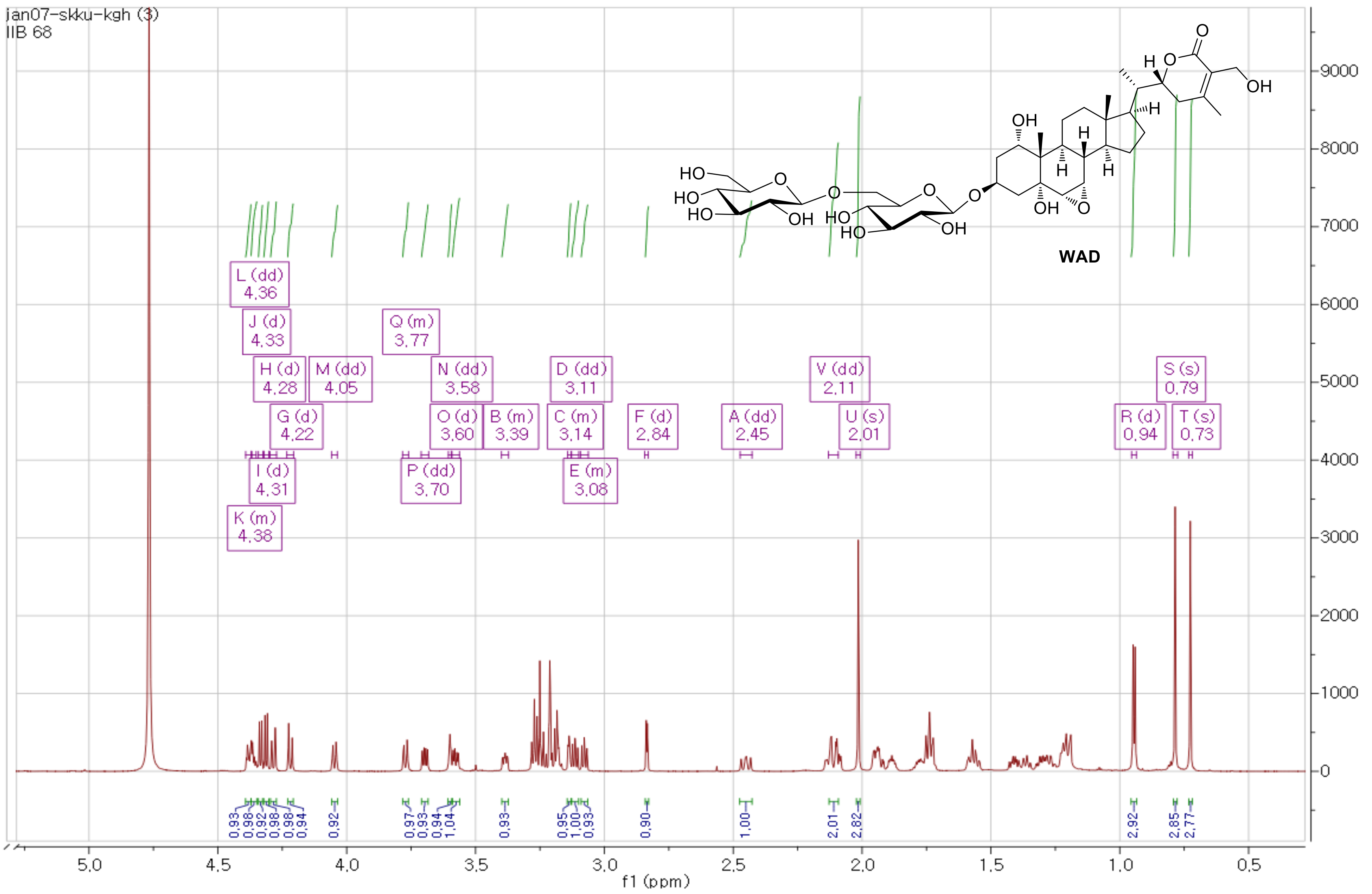

Figure S1. ^{1}H NMR (850 MHz) data for WAD in CD_3OD
